# Geographical disparities in maternal healthcare and mortality in the Philippines: a 10-year comparison

**DOI:** 10.1093/inthealth/ihaf059

**Published:** 2025-05-29

**Authors:** Francis Sarial Ganancial, Roditt Cruz-Delfino, Vivian Chia-Rong Hsieh

**Affiliations:** Department of Public Health, China Medical University, Taichung 406040, Taiwan; Graduate School, Notre Dame of Marbel University, City of Koronadal 9506, South Cotabato, Philippines; Department of Public Health, China Medical University, Taichung 406040, Taiwan; Department of Health Services Administration, China Medical University, Taichung 406040, Taiwan

**Keywords:** geographical disparities, maternal health, maternal health service utilization, maternal mortality, Philippines

## Abstract

**Background:**

Although the Philippines launched its Maternal, Neonatal, and Child Health and Nutrition strategy in 2008 to reduce maternal mortality, its impact on maternal health services (MHS) utilization and maternal mortality ratio (MMR) remains unclear. This study examines MHS utilization trends and their association with MMR across the Philippines from 2010 to 2019.

**Methods:**

We conducted an ecological study using panel data in the Philippines during 2010–2019. Secondary analyses took place on datasets from the Department of Health and the Philippine Statistics Authority. Multivariate analyses were performed to investigate the association between MHS utilization and MMR.

**Results:**

From 2010 to 2019, national mean MMR declined from 74.85 to 57.19 deaths per 100 000 live births. Facility-based deliveries increased by 39.57% and births attended by medical doctors by 19.01%. Results from fixed-effects models revealed no significant association between MHS utilization and change in MMR over the 10-y period. However, utilization of antenatal care in Luzon (β=−1.528, p=0.005) and postpartum care in Mindanao (β=−1.604, p=0.020) was attributable to MMR reduction in 2019.

**Conclusions:**

While a national decline in MMR was observed from 2010 to 2019, significant regional disparities in MMR existed. The impact of MHS utilization varied over time and location.

## Introduction

Maternal health constitutes the overall health and well-being of women during pregnancy and childbirth.^1^ It provides a continuum of healthcare, including family planning, preconception, antenatal, intrapartum and postpartum care, to reduce maternal morbidity and mortality.^2^ Preventable causes related to pregnancy and childbirth claim the lives of approximately 800 women around the world every day, equating to one death every 2 min.^3^ Hemorrhage, sepsis, hypertensive disorders, delivery complications, unsafe abortions and embolism are the primary causes of maternal deaths; however, these conditions can be effectively addressed through early detection and access to quality maternal health services (MHS).^4,5^ Beyond known medical causes, the 253 000 maternal deaths recorded in 2020 were largely attributable to inadequate healthcare systems, social determinants (including socioeconomic status, education and gender inequality) and external factors such as crises, particularly in developing countries.^5^ Maternal mortality still remains a significant public health issue on the global agenda.^5–7^ Countries around the world have included maternal health as an important target in both the Millennium Development Goals in 2000 and the Sustainable Development Goals (SDGs) in 2015, which highlighted the importance of quality MHS provision for improving population health outcomes.^6–10^

Over the years, global efforts have been directed toward improving the uptake of MHS.^6,7^ Global average rates indicate high utilization for several key services: antenatal care (ANC) (88% for at least one visit, 75% for four or more visits),^8^ skilled birth attendance (86%), facility-based delivery (FBD; 80%) and postpartum care (62%).^9^ These statistics highlight collective global initiatives aimed at improving overall maternal health^10^ and achieving international health targets.^7^

Maternal health care is a crucial part of healthcare in the Philippines, and the government, through the Department of Health (DOH), has implemented numerous policies and programs to address maternal health concerns since the early 1970s.^11–13^ These efforts have included law enactments, policy changes, public health campaigns and community-based interventions aimed at improving maternal health outcomes.^12,13^

In 2008, the Administrative Order 2008–0029, or ‘Implementing Health Reforms for Rapid Reduction of Maternal and Neonatal Mortality’, was introduced to provide an integrated Maternal, Newborn and Child Health, and Nutrition (MNCHN) strategy for the Filipino people.^14^ This strategy consists of interventions targeting all pregnant women throughout the care continuum, including ANC, skilled birth attendance, FBD, postpartum care and family planning.^15^ It was further reviewed and expanded to the Reproductive Health, Maternal-Newborn, Child, Adolescent Health, and Nutrition program based on the new guidelines on intrapartum care for positive childbirth in 2018.^16^ One of its objectives was to provide universal access to and utilization of MHS components and interventions for all. Uniformly implemented across the Philippines since 2008, it guides all eligible pregnant women in accessing and receiving the necessary maternal services specified in the program protocol, regardless of their location, socioeconomic status, age, ethnicity or religion.^15^ Consequently, regional variations in conditions and constraints led local governments to tailor MNCHN strategies to their contexts.^15,16^

Several studies in the Philippines and other countries have emphasized the importance of access to quality MHS across the maternal care continuum, from antenatal^17–19^ to postpartum care.^20^ However, only a few studies in the Philippines have investigated the impact of MHS utilization on reducing maternal deaths across geographical regions following implementation of interventions. Therefore, this study aimed to explore the temporal trends of change in maternal mortality ratio (MMR) and MHS utilization rate from 2010 to 2019, and to investigate the association between MHS utilization and MMR in the three main island groups of the Philippines.

## Methods

### Data source and sample size

This secondary data analysis used population-based data for a total of 120 cities^a^ and 81 provinces in the Philippines from 2010 to 2019. Only cities with incomplete data throughout the study period were excluded from the study (n=26). City- and provincial-level data from the three main islands were collected and aggregated to regional means, as presented in the Results. Data were obtained and downloaded from open public sources that belong to the respective archives of the DOH and the Philippine Statistics Authority. We collected data for MHS utilization and density of human resources for health (HRH) from the significant chapters of the Field Health Service Information System (FHSIS) annual report.^21,22^ The Census of Population and Housing provided data on the level of urbanization of the country.^23,24^

### Outcome variable

Maternal health outcome was measured using the MMR. The MMR is uniformly defined as the number of maternal deaths during pregnancy or within 42 d after delivery per 100 000 live births, irrespective of the duration and the site of the pregnancy, from any cause related to or aggravated by the pregnancy or its management.^5,25^ Accidental or incidental causes of death of pregnant women were not included as maternal deaths. This is consistent with the definition of maternal deaths adopted by the Filipino authorities.^15^

### Explanatory variables

The main explanatory variables of the study included the utilization of MHS, including ANC, FBD, births attended by skilled professionals (stratified into medical doctors, public health nurses and registered midwives) and postpartum care. The MNCHN strategy^15^ specifies the delivery of these MHS for each stage of conception through to, and after, birth.

Based on the FHSIS,^21^ ANC utilization was measured as a percentage of pregnant women who had at least four ANC visits. FBDs represent the proportion of births delivered in health facilities. Birth attendance by skilled professionals was derived from the percentage of deliveries attended by medical doctors, public health nurses or registered midwives. Postpartum care was measured by the proportion of postpartum women completing at least two postnatal check-ups. All explanatory variables were continuous and expressed as percentages to quantify the levels of service utilization at the regional level.

### Covariates

Data on HRH were extracted from the 2010 and 2019 FHSIS annual reports.^21,22^ This study used medical doctors, public health nurses, registered midwives and barangay health workers^b^ per 10 000 population, with all variables treated as continuous. The level of urbanization,^26^ or the percentage of population residing in urban areas across the Philippines, was also included as a continuous variable. Other HRH (e.g. sanitary personnel, nutritionists and medical technologists) were not included in this study due to the lack of distinct connections in MHS provision.

### Statistical analyses

Descriptive statistics were used to summarize the data. Means and SDs were calculated for all continuous variables. For bivariate analysis, Spearman correlation coefficients were used to measure the correlation of each explanatory variable with MMR. For multivariate analyses, fixed-effects models and generalized linear models were applied to examine the impact of MHS utilization on MMR during the study period while controlling the other covariates. The population size of each region was weighted in the multivariate analysis. Multi-collinearity was assessed using the variance inflation factor before the multivariate analyses to examine correlations between the explanatory variables.

Stratified analyses by the three main island groups (Luzon, Visayas and Mindanao) were later performed to explore regional variations within the Philippines. QGIS version 3.34.0 (QGIS Development Team, Open Source Geospatial Foundation Project, Chicago, IL, USA) was used to map the geospatial distribution and mean MMR of the country. All analyses were performed using SAS statistical software package version 9.4 (SAS Institute Inc., Cary, North Carolina, USA). p<0.05 was considered statistically significant.

## Results

### Sample characteristics and distribution

This study included 201 provinces and cities (81 provinces, 40.3%; 120 cities, 59.7%), grouped into 17 administrative regions and the three main islands of Luzon, Visayas and Mindanao (Table S1). The island of Luzon (51.74%; 104/201) has the highest representation of cities and provinces among all island groups. Approximately 23.08% of the cities and provinces in Luzon were from the Cavite, Laguna, Batangas, Rizal and Quezon (CALABARZON) region (24/104). Twenty-two cities and provinces in the Western Visayas region (45.83%) largely represented the Visayas island. More than one-quarter (28.57%; 14/49) of the samples in Mindanao were from the Northern Mindanao region. A snapshot of the administrative divisions and geographical characteristics of the country is presented in Figure S1.

### Change in MMR between 2010 and 2019

**Figure 1.**
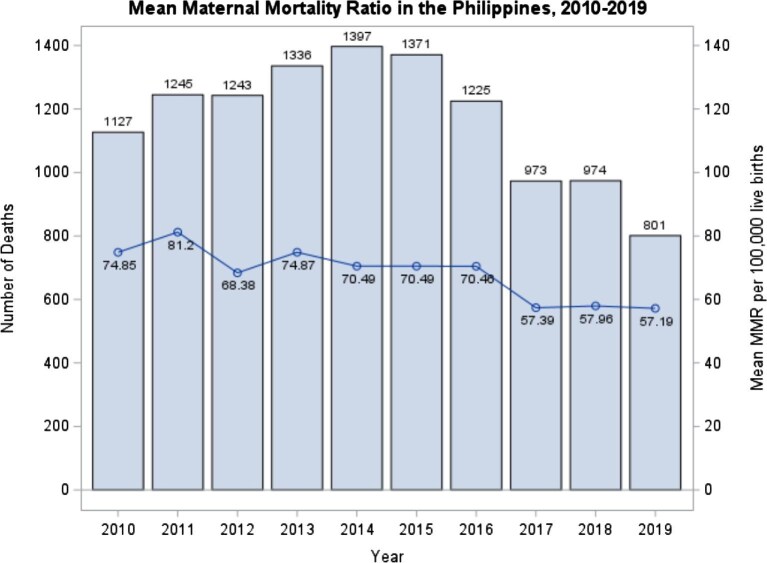
Mean maternal mortality in the Philippines from 2010 to 2019. MMR: maternal mortality ratio.

**Figure 2.**
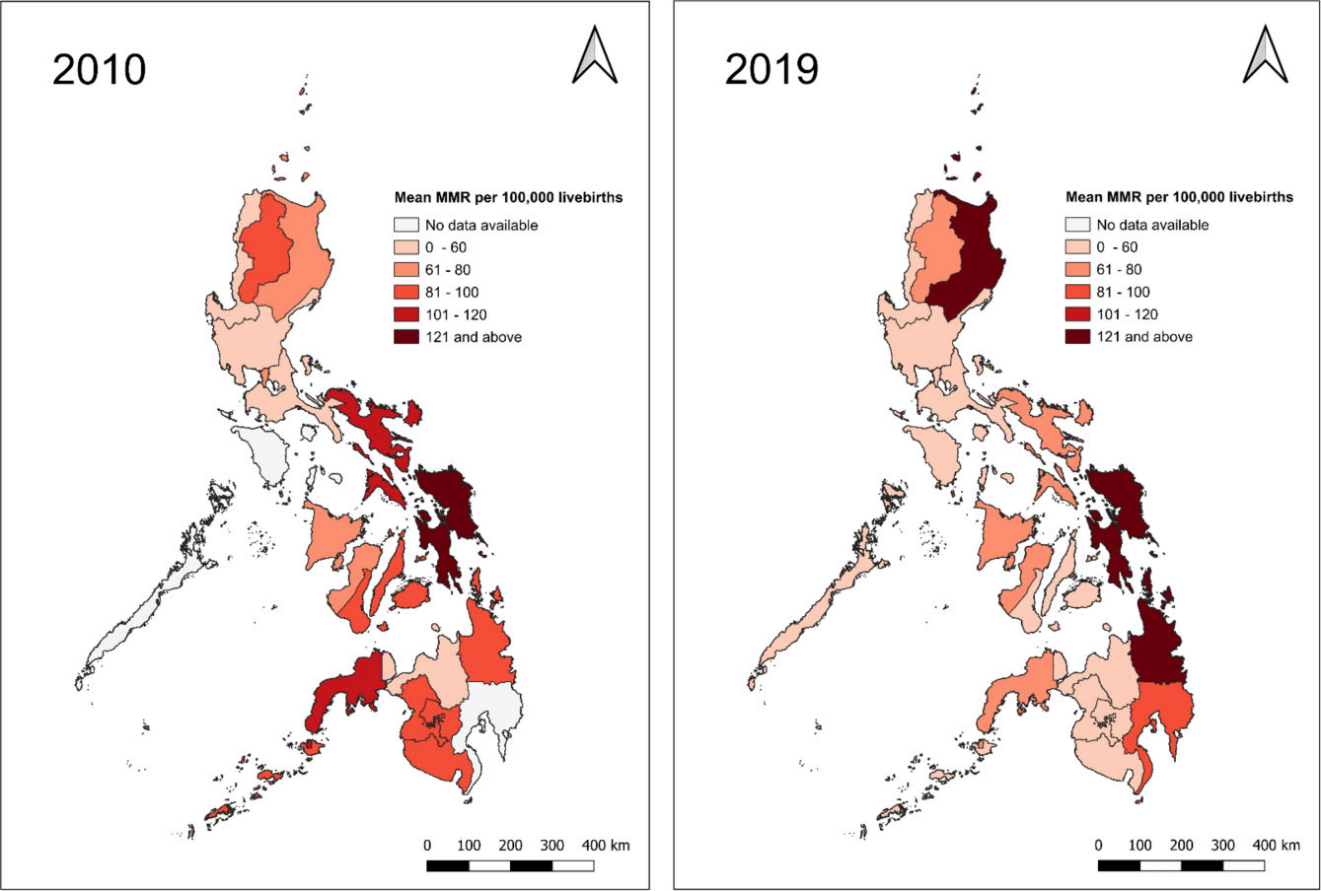
Geographical distribution of the mean maternal mortality ratios (MMRs) in the 17 administrative regions of the Philippines in 2010 (left) and 2019 (right). Generated from QGIS (version 3.34.0) with NAMRIA (National Mapping and Resource Information Authority, the Philippine government's central mapping agency) as base map.

**Figure 3.**
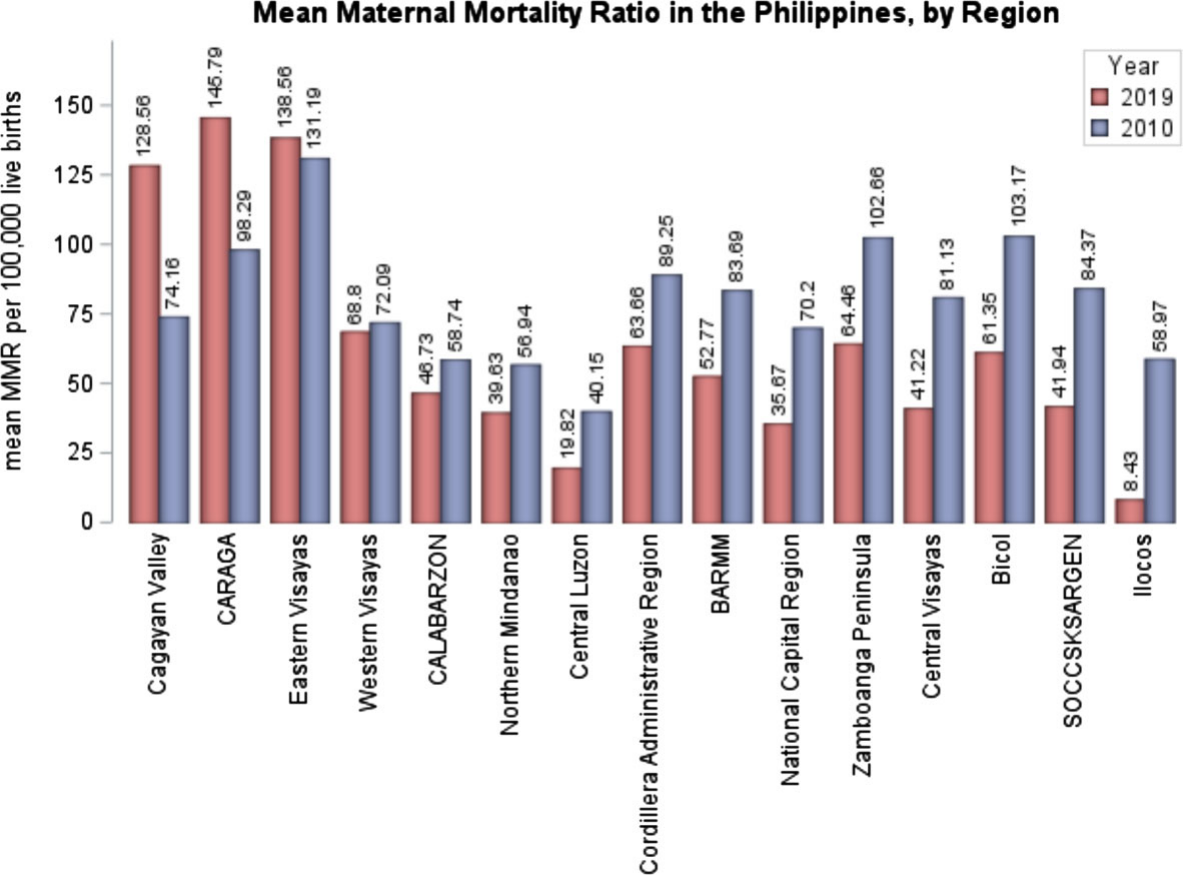
Changes in mean MMR in the Philippines by administrative region, 2010 vs 2019. Note: regions of Davao and MIMAROPA were excluded due to incomplete data for the year 2010. BARMM: Bangsamoro Autonomous Region in Muslim Mindanao; CALABARZON: Cagayan, Laguna, Batangas, Rizal, and Quezon; MIMAROPA: Mindoro, Marinduque, Romblon, and Palawan; NCR: National Capital Region; MMR: maternal mortality ratio; SOCCSKSARGEN: South Cotabato, Cotabato, Sultan Kudarat, Sarangani, and General Santos City.

As illustrated in Figure 1, the country's mean MMR decreased from 74.85/100 000 in 2010 to 57.19/100 000 in 2019, a moderate reduction of 17.66/100 000. When comparing the geographical distribution of mean MMR between 2010 and 2019, it is evident that, despite a notable overall decline in mean MMR over the 10-y period, regions such as Luzon and Mindanao exhibit increased MMRs (Figure 2). Specifically, in regions such as Cagayan Valley and Caraga, the MMR increased by 54.4 deaths per 100 000 live births (from 74.16 to 128.56) and 47.5 deaths per 100 000 live births (from 98.29 to 145.79), respectively, between 2010 and 2019 (Figure 3). Contrarily, the regions of Ilocos and SOCCSKSARGEN^c^ demonstrated the largest decline in maternal deaths, by 50.54 deaths per 100 000 (from 58.97 to 8.43) and 42.43 deaths per 100 000 (from 84.37 to 41.94), respectively, from 2010 to 2019 (Table 1). Trends in MMR for all 17 administrative regions from 2010 to 2019 are shown in Figure S2 to Figure S4.

**Table 1.** Comparison of mean maternal mortality ratio (per 100 000 live births) between 2010 and 2019 by island group and region

	2010		2019		Difference	
	(a)		(b)		(b—a)	
Island group/region	Mean	SD	Mean	SD	Mean	SD
**All**	**74.85**	74.52	**57.19**	88.42	**−17.66**	13.9
Luzon						
NCR (n=16)	**70.20**	63.20	**35.67**	24.99	**−34.53**	−38.21
CAR (n=7)	**89.25**	95.23	**63.66**	12.34	**−25.60**	−82.89
Ilocos (n=12)	**58.97**	48.78	**8.43**	29.21	**−50.54**	−19.57
Cagayan Valley (n=9)	**74.16**	111.12	**128.56**	252.15	**54.40**	141.03
Central Luzon (n=21)	**40.15**	31.08	**19.82**	37.64	**−20.33**	6.56
CALABARZON (n=24)	**58.74**	50.83	**46.73**	62.22	**−12.01**	11.39
MIMAROPA (n=6)	**-**	-	**21.69**	28.26	**-**	-
Bicol (n=9)	**103.17**	36.6	**61.35**	72.57	**−41.82**	35.97
Visayas						
Western Visayas (n=22)	**72.09**	79.04	**68.8**	61.44	**−3.69**	−17.6
Central Visayas (n=21)	**81.13**	89.07	**41.22**	38.5	**−39.91**	−50.57
Eastern Visayas (n=10)	**131.19**	150.62	**138.56**	140.89	**7.37**	−9.73
Mindanao						
Zamboanga Peninsula (n=8)	**102.66**	87.3	**64.46**	38.04	**−38.20**	−49.26
Northern Mindanao (n=14)	**56.94**	53.03	**39.63**	43.62	**−17.31**	−9.41
Davao (n=6)	**-**	-	**100.51**	50.14	**-**	-
SOCCSKSARGEN (n=6)	**84.37**	48.35	**41.94**	54.81	**−42.43**	6.46
Caraga (n=8)	**98.29**	64.31	**145.79**	133.91	**47.50**	69.6
BARMM (n=7)	**83.69**	46.92	**52.77**	55.88	**−30.92**	8.96

BARMM: Bangsamoro Autonomous Region in Muslim Mindanao; CALABARZON: Cagayan, Laguna, Batangas, Rizal, and Quezon; CAR: Cordillera Administrative Region; MIMAROPA: Mindoro, Marinduque, Romblon, and Palawan; NCR: National Capital Region; SOCCSKSARGEN: South Cotabato, Cotabato, Sultan Kudarat, Sarangani, and General Santos City.

Note: - : no data available.

### MHS utilization

Tables S2 and S3 summarize regional MHS utilization levels for 2010 and 2019, respectively. Among the MHS components, FBDs showed the greatest improvement, increasing from 51.90±22.95% in 2010 to 91.47±23.85% in 2019. Births attended by medical doctors also increased significantly (from 42.44±32.95% to 61.45±30.63%), as did postpartum care coverage, although more moderately (from 54.93±15.57% to 62.76±22.46%). ANC utilization moderately increased by 2.31% (from 58.45% to 60.76%), while births attended by public health nurses remained constant. However, births attended by registered midwives declined by 7.39% (from 38.16% to 30.77%).

Comparing island groups, Mindanao and Luzon demonstrated more substantial improvements in FBD utilization over the 10-y period than Visayas (+43.42% and +42.49% vs +32.92%, respectively). Mindanao also experienced the largest increase in births attended by medical doctors (+25.51% compared with +20.23% in Visayas and +15.59% in Luzon) and a moderate rise in ANC utilization (+3.25% vs +4.43% in Visayas and +0.83% in Luzon). Correlation coefficients between study variables can be found in Tables S4 to S6.

### Association between MHS utilization and MMR

The association between MMR and MHS utilization across all regions over the 10-y period was examined using fixed-effects models, as presented in Table S7. When only MHS components were considered in Model 1, higher FBD was significantly associated with a lower MMR (β=−0.415, p=0.045). However, this association diminished in Models 2 and 3 after adjusting for HRH and then for urbanization level, respectively. Overall, the fixed-effects models indicated no significant relationship between any of the explanatory variables and the change in MMR across all Philippine regions over the study period.

Table 2 presents the results of generalized linear regression models for 2010 and 2019. Model 1 indicated that, in 2010, births attended by medical doctors were positively associated with MMR (β=0.414, p=0.043), while all other MHS variables were not statistically significant. However, in 2019, the effect of births attended by medical doctors was no longer statistically significant, while ANC provision became negatively associated with the outcome (β=−0.856, p=0.033). In Model 2, the density of public health nurses was found to be positively associated with MMR in 2010 (β = 31.988, p < 0.0001), while ANC was still negatively associated with MMR in 2019 (β=−0.798, p=0.049). In the full model after adjusting for all covariates, Model 3 revealed that births attended by medical doctors and density of public health nurses were associated with higher MMR in 2010 (β=0.398, p=0.049 and β=31.983, p<0.0001, respectively). In 2019, the negative association between ANC and MMR persisted (β=−0.832, p=0.040), while births attended by public health nurses were associated with increased maternal deaths (β=6.075, p=0.046).

**Table 2.** Generalized linear model analysis on the association between maternal health service utilization and maternal mortality ratio in the Philippines, 2010 and 2019

	2010						2019					
	Model 1		Model 2		Model 3		Model 1		Model 2		Model 3	
Variables	β	p	β	p	β	p	β	p	β	p	β	p
Maternal health services												
Antenatal care	**0.139**	0.736	**0.257**	0.532	**0.233**	0.578	**−0.856**	0.033	**−0.798**	0.049	**−0.832**	0.040
Facility-based delivery	**−0.258**	0.348	**−0.305**	0.263	**−0.291**	0.292	**0.167**	0.740	**0.146**	0.770	**0.147**	0.769
Births attended by medical doctor	**0.414**	0.043	**0.393**	0.051	**0.398**	0.049	**0.157**	0.769	**0.183**	0.733	**0.101**	0.850
Births attended by public health nurse	**3.362**	0.177	**2.541**	0.294	**2.477**	0.309	**5.875**	0.051	**5.413**	0.072	**6.075**	0.046
Births attended by registered midwife	**−0.238**	0.505	**−0.189**	0.587	**−0.179**	0.608	**−0.362**	0.530	**−0.281**	0.626	**−0.370**	0.522
Postpartum care	**−0.765**	0.189	**−0.797**	0.164	**−0.770**	0.184	**0.503**	0.245	**0.443**	0.311	**0.359**	0.414
Human resources for health												
Medical doctor			**−35.625**	0.178	**−34.579**	0.196			**−40.722**	0.260	**−51.916**	0.160
Public health nurse			**31.988**	<0.001	**31.983**	<0.001			**−0.521**	0.932	**0.172**	0.977
Registered midwife			**−4.406**	0.379	**−4.665**	0.359			**5.169**	0.520	**6.737**	0.405
Barangay health worker			**0.213**	0.508	**0.167**	0.638			**0.551**	0.321	**0.820**	0.143
Socioeconomic factor												
Urbanization level					**−0.062**	0.754					**0.383**	0.159
Observations		174		174		174		178		178		178
R-square		0.074		0.150		0.151		0.077		0.104		0.115

### Utilization of MHS and MMR by island group

Table 3 presents multivariate analyses stratified by the three main island groups, showing only results from the full models. In 2010, births attended by medical doctors in Luzon were found to be negatively and significantly associated with the magnitude of MMR (β=−1.329, p=0.039). Meanwhile, the density of public health nurses in Luzon (β=27.668, p=0.002) and Visayas (β=62.031, p = 0.047) was statistically significant but positively associated with MMR.

**Table 3.** Generalized linear model analysis on the association between maternal health service utilization and maternal mortality ratio in the Philippines by island group, 2010 and 2019

	Luzon				Visayas				Mindanao			
	2010		2019		2010		2019		2010		2019	
Variables	β	p	β	p	β	p	β	p	β	p	β	p
Maternal health services												
Antenatal care	**0.131**	0.789	**−1.528**	0.005	**−0.436**	0.739	**−0.717**	0.812	**1.266**	0.170	**0.615**	0.342
Facility-based delivery	**0.626**	0.200	**0.183**	0.726	**−0.885**	0.253	**1.959**	0.825	**1.035**	0.084	**−1.370**	0.326
Births attended by medical doctor	**−1.329**	0.039	**−0.072**	0.896	**0.526**	0.099	**−0.952**	0.879	**−0.670**	0.612	**0.302**	0.854
Births attended by public health nurse	**2.256**	0.376	**7.903**	0.019	**−0.267**	0.983	**1.627**	0.955	**−2.509**	0.691	**−10.412**	0.125
Births attended by registered midwife	**−0.890**	0.135	**0.353**	0.556	**0.009**	0.990	**−1.872**	0.777	**1.158**	0.208	**−0.010**	0.995
Postpartum care	**−0.483**	0.469	**1.303**	0.033	**−1.660**	0.315	**−2.625**	0.500	**−2.411**	0.156	**−1.604**	0.020
Human resources for health												
Medical doctor	**−9.790**	0.820	**−21.686**	0.609	**−87.186**	0.101	**−339.288**	0.277	**18.597**	0.872	**−129.848**	0.203
Public health nurse	**27.668**	0.002	**−2.649**	0.787	**62.031**	0.047	**−10.733**	0.641	**−15.326**	0.632	**21.487**	0.060
Registered midwife	**−2.633**	0.688	**8.481**	0.388	**−29.642**	0.069	**24.586**	0.734	**−7.915**	0.560	**−1.295**	0.941
Barangay health worker	**0.630**	0.241	**0.856**	0.245	**−0.656**	0.483	**0.276**	0.906	**−0.507**	0.664	**0.859**	0.376
Socioeconomic factor												
Urbanization level	**0.258**	0.292	**0.367**	0.342	**−1.046**	0.098	**1.235**	0.597	**0.660**	0.292	**0.648**	0.158
Observations		88		104		48		25		38		49
R-square		0.291		0.213		0.415		0.464		0.356		0.353

In 2019, there were notable changes in the trend over the 10-y period. The results revealed that an increase in utilization of ANC in Luzon (β=−1.528, p=0.005) and postpartum care in Mindanao (β=−1.604, p=0.020) had a significant negative relationship with MMR. On the other hand, in Luzon, births attended by public health nurses (β=7.903, p=0.019) and postpartum care utilization (β=1.303, p=0.033) were associated with increased maternal deaths. No explanatory variable showed statistical significance for the Visayas island group.

## Discussion

### MMR trends from 2010 to 2019

Nationwide trends revealed an overall improvement in maternal health, where a remarkable reduction in the MMR was observed from 2010 to 2019 in the Philippines. This positive trend indicates that the 2030 SDG target (70 maternal deaths per 100 000 live births) has been achieved.^7^ Several factors likely contributed to the marked improvement in MMR, including the concerted efforts of healthcare providers (e.g. delivery of curative and preventative health services, home visitations, community-based education, micronutrient supplementation, immunization and antenatal and postpartum check-ups^27^) and advancements in health infrastructure.^28^

Despite the observed national decline in MMR, significant regional variations in its geographical distribution were evident between 2010 and 2019, with regions exhibiting both the greatest declines and increases located within the same island groups (e.g. Ilocos and Cagayan Valley in Luzon; SOCCSKSARGEN and Caraga in Mindanao). Preventable maternal deaths are a consequence of biomedical complications and many other factors, including those beyond health systems, such as systematic inequities in socioeconomic development, which vary by geographical context.^29^ These inequities may have manifested as regional variations in the MMR, influenced by cultural, political and economic systems, data for which are unfortunately unavailable for examination.

### MHS utilization and MMR

Utilization of MHS also showed diverse patterns. For instance, proportions of FBDs and doctor-attended births greatly increased over the years, but their utilization rates varied regionally. A previous study highlighted regional disparities in healthcare access across the three main islands of the Philippines.^30,31^ It was indicated that healthcare development has been centralized in Luzon, particularly the National Capital Region,^30^ creating inequities in utilization of health services due to uneven development across the country. This regional heterogeneity likely explains the lack of a significant correlation between changes in MHS utilization and MMR reduction at the national level over the 10-y period.

However, we observed that an increase in utilization of ANC was negatively and statistically associated with MMR after adjusting for all covariates in 2019. This finding supports the recommendation of at least four ANC visits as a key intervention to reduce MMR by facilitating early risk identification, preventing and managing pregnancy-related complications, and providing essential health education and counseling.^8,32^ In many low- and middle-income countries, the implementation of ANC interventions has been associated with reductions in MMR and improved maternal health outcomes, such as Bangladesh,^33^ China,^34,35^ India,^18^ Indonesia,^19^ Kenya^18^ and Pakistan.^18,36^ A systematic review conducted in sub-Saharan Africa found that adequate ANC improves the odds of FBD, which, in return, significantly lowers maternal mortality risks.^37^

Still, the association between MHS utilization and MMR varied across Luzon, Visayas and Mindanao. Unlike the Visayas island, the more developed islands of Luzon and Mindanao demonstrated positive effects of specific MHS components, such as ANC and doctor-attended births, on MMR, in both 2010 and 2019. This finding further supports our argument that maternal health is influenced not only by access to MHS and health system development, which vary significantly across regions, but also by non-biomedical factors specific to each geographical setting. The lack of a significant association between MMR and MHS uptake in the Visayas suggests that these other factors may be exerting a greater influence on MMR.^29^

While this study demonstrated the vital role of ANC in mitigating maternal mortality, the effectiveness of other MHS interventions is less clear. Specifically, birth attendance by medical doctors and public health nurses showed contrasting or statistically insignificant effects, suggesting that factors other than utilization, such as quality of care and healthcare workforce training, may be crucial.^34,38^ Should this information become available, assessment of birth attendants’ ability to provide adequate care during delivery will be essential.

### Study limitations and strengths

This study has several limitations. First, the limited type and amount of available data constrained our sample size and may have led us to overlook many more contributing factors to MMR. Second, the study design does not allow us to establish causal relationships between the variables examined. Finally, the study's reliance on aggregated data from public and government health institutions precluded analysis of individual-level variations in MHS and MMR. Future studies should consider other contributory factors, such as the quality of MHS, socioeconomic status and structural determinants, which can affect maternal morbidity and mortality.

The strengths of this study lie in its long study period of 10 y and the use of a nationally representative dataset, which enabled us to conduct a comprehensive assessment of the association between MHS utilization and MMR across the various regions of the Philippines, including stratified analysis by island group. To the best of our knowledge, no previous study has conducted such analysis, and related evidence remains limited.

### Policy implications

Because limited studies have investigated the association of MHS utilization and MMR across the Philippines, this study aimed to address this gap by incorporating city- and provincial-level data. Analyzing geographical variations reveals how factors beyond service utilization influence the impact of MHS interventions. While the MNCHN strategy aims for universal access to and utilization of MHS interventions, local government units must contextualize their strategies to account for regional variations. Targeted and varied interventions are needed to address the unique challenges faced by pregnant women, especially those in less developed regions, to further reduce disparities in maternal healthcare and outcomes. These interventions may include improving health infrastructure, expanding community-based education, strengthening referrals and ensuring universal access to quality MHS, as seen in the more developed regions.^12,13,16,30,31^

### Conclusions

Although remarkable strides have been made in maternal health outcomes and MHS utilization in the Philippines over the 10-y period, significant geographical gaps in service uptake and MMR are evident across administrative regions. This likely explains the lack of correlation between changes in MHS utilization and MMR reduction at the national level. While ANC and doctor-attended births demonstrated positive effects on MMR, their impact was not uniformly distributed across the country. Context-specific MNCHN strategies need to be considered to account for the regional variations in development and other structural determinants.

## Supplementary Material

ihaf059_Supplemental_File

## Data Availability

Aggregated raw data used in this study are available from the corresponding author upon reasonable request.

## Footnotes

a Among the 146 cities, only 120 were included in this study due to data availability from the FHSIS annual reports, while data from all 81 provinces in the country were used for analysis.

b Barangay health workers are accredited community-based volunteers in the Philippine healthcare system who provide frontline primary healthcare services, including maternal health support and health education, within their villages and/or communities (i.e. barangays).^16^

c SOCCSKSARGEN is a region and an acronym derived from the first syllables or letters of its component provinces and city: South Cotabato, Cotabato, Sultan Kudarat, Sarangani, and General Santos City.
