# Supplementary material for: Geographical disparities in maternal healthcare and mortality in the Philippines: a 10-year comparison
Source: Int Health. 2025 May 29;18(1):83–92. doi: 10.1093/inthealth/ihaf059 (PMC12766459; doi:10.1093/inthealth/ihaf059)
Supplement: ihaf059_Supplemental_File [file ihaf059_supplemental_file.docx]

**Supplementary Materials**

**Geographical disparities in maternal healthcare and mortality in the Philippines: a 10-year comparison**

Authors: Francis S. Ganancial, Roditt C. Delfino and Vivian Chia-Rong Hsieh

**Tables:** Table S1 to Table S7.

**Figures:** Figure S1 to Figure S4.

**Supplementary Tables**

**Table S1. Sample characteristics and distribution**

|  | **Province**  **(n = 81)** | **City**  **(n = 120)** | **All**  **(n=201)** |
| --- | --- | --- | --- |
|  | n (%) | n (%) | n (%) |
| **Luzon** | **38 (46.91)** | **66 (55.0)** | **104 (51.74)** |
| 1 National Capital Region (NCR) | 0 (0) | 16 (24.24) | 16 (15.38) |
| 2 Cordillera Administrative Region (CAR) | 6 (15.79) | 1 (1.52) | 7 (6.73) |
| 3 Ilocos | 4 (10.53) | 8 (12.12) | 12 (11.54) |
| 4 Cagayan Valley | 5 (13.16) | 4 (6.06) | 9 (8.65) |
| 5 Central Luzon | 7 (18.42) | 14 (21.21) | 21 (20.19) |
| 6 Cavite, Laguna, Batangas, Rizal, and Quezon (CALABARZON) | 5 (13.16) | 19 (28.79) | 24 (23.08) |
| 7 Mindoro, Marinduque, Romblon, and Palawan (MIMAROPA) | 5 (13.16) | 1 (1.52) | 6 (5.77) |
| 8 Bicol | 6 (15.79) | 3 (4.55) | 9 (8.65) |
| **Visayas** | **16 (19.75)** | **32 (26.67)** | **48 (23.88)** |
| 9 Western Visayas | 6 (37.50) | 16 (50.0) | 22 (45.83) |
| 10 Central Visayas | 4 (25.0) | 12 (37.50) | 16 (33.33) |
| 11 Eastern Visayas | 6 (37.50) | 4 (12.50) | 10 (20.83) |
| **Mindanao** | **27 (33.33)** | **22 (18.33)** | **49 (24.38)** |
| 12 Zamboanga Peninsula | 3 (11.11) | 5 (22.73) | 8 (16.33) |
| 13 Northern Mindanao | 5 (18.52) | 9 (40.91) | 14 (28.57) |
| 14 Davao | 5 (18.52) | 1 (4.55) | 6 (12.24) |
| 15 South Cotabato, Cotabato, Sultan Kudarat, Sarangani, and General Santos City (SOCCSKSARGEN) | 4 (14.81) | 2 (9.09) | 6 (12.24) |
| 16 Caraga | 5 (18.52) | 3 (13.64) | 8 (16.33) |
| 17 Bangsamoro Autonomous Region in Muslim Mindanao (BARRM) ^a^ | 5 (18.52) | 2 (9.09) | 7 (14.29) |

Note: ^a^ - Renamed region from ARMM under Republic Act No.11054, dated July 27, 2018; ratified on January 25, 2019.

**Table S2. Utilization of maternal health services across different regions in the Philippines, 2010**

| **Island group / Region** | n | **Antenatal Care** | | **Facility-based delivery** | | **Skilled birth attendance** | | | | | | **Postpartum care** | |
| --- | --- | --- | --- | --- | --- | --- | --- | --- | --- | --- | --- | --- | --- |
|  |  |  |  |  |  | **Medical doctor** | | **Public health nurse** | | **Registered midwife** | |  |  |
|  |  | **Mean** | SD | **Mean** | SD | **Mean** | SD | **Mean** | SD | **Mean** | SD | **Mean** | SD |
| **All** | **201** | **58.45** | 21.48 | **51.90** | 22.95 | **42.44** | 32.95 | **1.19** | 2.26 | **38.16** | 18.07 | **54.93** | 15.57 |
|  |  |  |  |  |  |  |  |  |  |  |  |  |  |
| **Luzon** | **104** | **60.19** | 22.18 | **49.71** | 19.53 | **47.09** | 19.68 | **1.14** | 2.53 | **37.19** | 17.17 | **56.96** | 15.49 |
| NCR | 16 | **51.39** | 22.74 | **50.46** | 21.11 | **53.01** | 19.81 | **0.49** | 0.46 | **36.06** | 16.74 | **50.94** | 18.73 |
| CAR | 7 | **47.29** | 16.37 | **63.29** | 20.09 | **57.70** | 22.22 | **1.99** | 1 | **25.10** | 18.37 | **44.82** | 14.72 |
| Ilocos | 12 | **78.50** | 19.79 | **58.91** | 23.16 | **59.47** | 20.65 | **2.35** | 5.96 | **31.88** | 16.46 | **65.77** | 18.03 |
| Cagayan Valley | 9 | **63.88** | 22.23 | **43.62** | 21.35 | **41.85** | 16.26 | **0.61** | 0.55 | **50.40** | 17.02 | **60.45** | 8.49 |
| Central Luzon | 21 | **63.99** | 19.12 | **52.19** | 17.66 | **48.45** | 17.63 | **0.36** | 0.79 | **40.41** | 18.63 | **60.74** | 15.36 |
| CALABARZON | 24 | **50.89** | 27.62 | **41.52** | 11.94 | **36.91** | 10.8 | **1.08** | 1.72 | **37.64** | 14.08 | **54.36** | 11.69 |
| MIMAROPA | 6 | **60.33** | 14.18 | **48.07** | 0.01 | **-** | - | **-** | - | **-** | - | **60.48** | 7.61 |
| Bicol | 9 | **65.10** | 9.23 | **41.07** | 24.26 | **31.53** | 20.57 | **2.32** | 2.37 | **36.01** | 15.21 | **56.14** | 14.76 |
| **Visayas** | **48** | **56.53** | 14.58 | **63.14** | 25.00 | **43.60** | 54.55 | **0.69** | 1.24 | **41.38** | 20.89 | **55.17** | 11.67 |
| Western Visayas | 22 | **55.50** | 12.64 | **69.81** | 17.07 | **49.58** | 78.37 | **0.37** | 0.6 | **42.86** | 25.36 | **55.55** | 8.40 |
| Central Visayas | 16 | **51.37** | 16.32 | **61.38** | 30.06 | **39.03** | 18.18 | **0.67** | 1 | **41.72** | 18.45 | **51.79** | 16.56 |
| Eastern Visayas | 10 | **67.04** | 10.98 | **51.27** | 28.39 | **37.76** | 22.3 | **1.44** | 2.18 | **37.59** | 13.79 | **59.76** | 6.76 |
| **Mindanao** | **49** | **56.85** | 25.89 | **44.17** | 23.42 | **32.12** | 18.49 | **1.80** | 2.41 | **36.61** | 16.46 | **50.44** | 18.50 |
| Zamboanga Peninsula | 8 | **74.22** | 22.39 | **32.56** | 17.94 | **30.69** | 19.64 | **2.23** | 2.21 | **44.56** | 20.65 | **58.36** | 6.51 |
| Northern Mindanao | 14 | **69.30** | 16.18 | **51.34** | 24.09 | **41.05** | 16.89 | **0.59** | 0.6 | **34.28** | 16.53 | **57.84** | 9.65 |
| Davao | 6 | **7.41** | 3.07 | **43.94** | 17.56 | **30.58** | 6.6 | **1.12** | 0.88 | **25.74** | 6.65 | **13.06** | 6.11 |
| SOCCSKSARGEN | 6 | **51.15** | 21.59 | **39.93** | 18.66 | **25.45** | 6.3 | **3.58** | 3.9 | **38.83** | 7.93 | **49.97** | 19.97 |
| Caraga | 8 | **54.71** | 15.95 | **67.26** | 18.42 | **44.36** | 19.94 | **2.07** | 2.46 | **29.84** | 17.6 | **53.14** | 14.15 |
| BARMM | 7 | **56.09** | 20.34 | **24.01** | 20.25 | **8.4** | 9.08 | **2.35** | 2.99 | **45.8** | 15.98 | **52.29** | 20.13 |

Note: SD – Standard deviation; - – no data available; NCR – National Capital Region; CAR – Cordillera Administrative Region; CALABARZON – Cagayan, Laguna, Batangas, Rizal, and Quezon; MIMAROPA – Mindoro, Marinduque, Romblon, and Palawan; SOCCSKSARGEN – South Cotabato, Cotabato, Sultan Kudarat, Saranggani, and General Santos City; BARMM - Bangsamoro Autonomous Region of Muslim Mindanao

**Table S3. Utilization of maternal health services across different regions in the Philippines, 2019**

| **Island group / Region** | n | **Antenatal Care** | | **Facility-based delivery** | | **Skilled birth attendance** | | | | | | **Postpartum care** | |
| --- | --- | --- | --- | --- | --- | --- | --- | --- | --- | --- | --- | --- | --- |
|  |  |  |  |  |  | **Medical doctor** | | **Public health nurse** | | **Registered midwife** | |  |  |
|  |  | **Mean** | SD | **Mean** | SD | **Mean** | SD | **Mean** | SD | **Mean** | SD | **Mean** | SD |
| **All** | **201** | **60.76** | 23.78 | **91.47** | 23.85 | **61.45** | 30.63 | **1.19** | 2.21 | **30.77** | 23.03 | **62.76** | 22.46 |
|  |  |  |  |  |  |  |  |  |  |  |  |  |  |
| **Luzon** | **104** | **61.02** | 26.15 | **92.20** | 29.04 | **62.68** | 34.91 | **1.12** | 2.55 | **30.22** | 25.32 | **62.84** | 24.21 |
| NCR | 16 | **71.72** | 23.32 | **91.05** | 24.45 | **75.25** | 15.82 | **0.54** | 0.74 | **21.95** | 14.35 | **82.03** | 24.81 |
| CAR | 7 | **58.06** | 15.14 | **95.55** | 2.82 | **86.12** | 10.46 | **2.11** | 1.38 | **9.37** | 9.27 | **65.99** | 18.14 |
| Ilocos | 12 | **67.22** | 15.04 | **99.52** | 0.85 | **90.59** | 11.22 | **0.14** | 0.18 | **9.18** | 10.99 | **60.21** | 10.42 |
| Cagayan Valley | 9 | **64.74** | 22.44 | **98.35** | 1.73 | **79.25** | 16.62 | **0.21** | 0.28 | **19.44** | 16.58 | **74.28** | 15.83 |
| Central Luzon | 21 | **69.59** | 34.07 | **76.40** | 28.91 | **27.80** | 32.2 | **0.64** | 2.38 | **50.09** | 30.34 | **55.72** | 29.89 |
| CALABARZON | 24 | **49.70** | 25.09 | **105.8** | 39.45 | **65.86** | 41.65 | **1.21** | 2.54 | **40.11** | 24.89 | **56.31** | 22.13 |
| MIMAROPA | 6 | **46.24** | 22.45 | **77.57** | 30.34 | **49.81** | 27.77 | **1.61** | 1.77 | **22.33** | 14.44 | **54.88** | 20.86 |
| Bicol | 9 | **53.38** | 25.26 | **86.13** | 32.37 | **49.83** | 26.58 | **4.20** | 5.57 | **32.44** | 21.34 | **57.70** | 24.35 |
| **Visayas** | **48** | **60.96** | 17.00 | **96.06** | 5.12 | **63.83** | 21.86 | **0.89** | 1.04 | **30.59** | 20.35 | **63.13** | 17.49 |
| Western Visayas | 22 | **54.08** | 14 | **95.44** | 3.23 | **27.18** | 30.44 | **0.46** | 4.44 | **28.66** | 28.8 | **59.22** | 10.63 |
| Central Visayas | 16 | **74.39** | 22.04 | **94.28** | 6.8 | **54.49** | 19.49 | **0.64** | 0.74 | **39.94** | 17.98 | **81.06** | 15.31 |
| Eastern Visayas | 10 | **57.06** | 9.78 | **97.82** | 4.97 | **67.69** | 14.36 | **1.41** | 1.37 | **25.60** | 12.01 | **53.71** | 14.48 |
| **Mindanao** | **49** | **60.10** | 21.75 | **87.59** | 15.73 | **57.63** | 24.17 | **1.49** | 1.85 | **32.04** | 19.24 | **62.39** | 21.18 |
| Zamboanga Peninsula | 8 | **55.58** | 25.88 | **91.61** | 11.39 | **57.94** | 28.63 | **2.34** | 1.91 | **32.17** | 25.75 | **40.69** | 8.61 |
| Northern Mindanao | 14 | **74.18** | 23.77 | **90.39** | 11.89 | **66.90** | 15.86 | **0.96** | 1.35 | **24.14** | 9.23 | **75.72** | 15.10 |
| Davao | 6 | **52.76** | 17.44 | **87.03** | 10.65 | **71.48** | 13.03 | **0.36** | 0.26 | **25.07** | 9.44 | **67.01** | 14.92 |
| SOCCSKSARGEN | 6 | **60.83** | 16.8 | **90.25** | 19.88 | **43.42** | 17.75 | **1.74** | 2.06 | **47.12** | 20.32 | **68.97** | 36.21 |
| Caraga | 8 | **55.73** | 11.67 | **94.97** | 4.69 | **70.74** | 16.84 | **1.49** | 2.15 | **22.94** | 15.34 | **61.91** | 13.77 |
| BARMM | 7 | **47.73** | 19.52 | **67.15** | 21.55 | **24.05** | 20.4 | **2.33** | 2.52 | **51.14** | 18.79 | **51.48** | 16.15 |

Note: SD – Standard deviation; - – no data available; NCR – National Capital Region; CAR – Cordillera Administrative Region; CALABARZON – Cagayan, Laguna, Batangas, Rizal, and Quezon; MIMAROPA – Mindoro, Marinduque, Romblon, and Palawan; SOCCSKSARGEN – South Cotabato, Cotabato, Sultan Kudarat, Saranggani, and General Santos City; BARMM - Bangsamoro Autonomous Region of Muslim Mindanao

**Table S4. Correlation between study variables, 2010–2019**

|  | **Variable** | **1** | **2** | **3** | **4** | **5** | **6** | **7** |
| --- | --- | --- | --- | --- | --- | --- | --- | --- |
| 1 | Maternal Mortality Ratio | 1 |  |  |  |  |  |  |
| 2 | Antenatal care | -0.118 | 1 |  |  |  |  |  |
| 3 | Facility-based delivery | -0.140 | 0.057 | 1 |  |  |  |  |
| 4 | Skilled birth attendance by Medical Doctor | -0.010 | 0.027 | 0.610 | 1 |  |  |  |
| 5 | Skilled birth attendance by Public Health Nurse | 0.183 | 0.005 | -0.162 | -0.116 | 1 |  |  |
| 6 | Skilled birth attendance by Registered Midwife | -0.052 | 0.066 | -0.301 | -0.712 | 0.124 | 1 |  |
| 7 | Postpartum care | -0.089 | 0.692 | 0.221 | 0.170 | -0.046 | 0.008 | 1 |

**Table S5. Correlation between study variables, 2010**

|  | **Variable** | **1** | **2** | **3** | **4** | **5** | **6** | **7** |
| --- | --- | --- | --- | --- | --- | --- | --- | --- |
| 1 | Maternal Mortality Ratio | 1 |  |  |  |  |  |  |
| 2 | Antenatal care | -0.094 | 1 |  |  |  |  |  |
| 3 | Facility-based delivery | -0.011 | -0.094 | 1 |  |  |  |  |
| 4 | Skilled birth attendance by Medical Doctor | -0.039 | -0.074 | 0.480 | 1 |  |  |  |
| 5 | Skilled birth attendance by Public Health Nurse | 0.170 | -0.036 | -0.285 | -0.222 | 1 |  |  |
| 6 | Skilled birth attendance by Registered Midwife | -0.120 | 0.222 | -0.283 | -0.564 | 0.065 | 1 |  |
| 7 | Postpartum care | -0.113 | 0.765 | -0.033 | -0.064 | 0.448 | 0.196 | 1 |

**Table S6. Correlation between study variables, 2019**

|  | **Variable** | **1** | **2** | **3** | **4** | **5** | **6** | **7** |
| --- | --- | --- | --- | --- | --- | --- | --- | --- |
| 1 | Maternal Mortality Ratio | 1 |  |  |  |  |  |  |
| 2 | Antenatal care | -0.113 | 1 |  |  |  |  |  |
| 3 | Facility-based delivery | -0.031 | 0.174 | 1 |  |  |  |  |
| 4 | Skilled birth attendance by Medical Doctor | 0.142 | 0.075 | 0.484 | 1 |  |  |  |
| 5 | Skilled birth attendance by Public Health Nurse | 0.201 | -0.017 | -0.178 | -0.068 | 1 |  |  |
| 6 | Skilled birth attendance by Registered Midwife | -0.081 | -0.026 | -0.202 | -0.813 | 0.099 | 1 |  |
| 7 | Postpartum care | 0.004 | 0.674 | 0.181 | 0.164 | -0.029 | -0.009 | 1 |

**Table S7. Fixed-effects analysis of maternal health service utilization and maternal mortality ratio in the Philippines, 2010-2019 (n=201)**

| **Variable** | **Model 1** | |  | **Model 2** | |  | **Model 3** | |
| --- | --- | --- | --- | --- | --- | --- | --- | --- |
|  | β | p-value |  | β | p-value |  | β | p-value |
| **Maternal health service utilization** |  |  |  |  |  |  |  |  |
| Antenatal care | **0.317** | 0.444 |  | **0.147** | 0.726 |  | **0.132** | 0.757 |
| Facility-based delivery | **-0.415** | 0.045 |  | **-0.261** | 0.237 |  | **-0.229** | 0.383 |
| Births attended by medical doctor | **0.522** | 0.049 |  | **0.521** | 0.049 |  | **0.501** | 0.072 |
| Births attended by public health nurse | **1.074** | 0.710 |  | **1.053** | 0.714 |  | **1.088** | 0.707 |
| Births attended by registered midwife | **0.444** | 0.271 |  | **0.471** | 0.249 |  | **0.455** | 0.274 |
| Postpartum care | **-0.581** | 0.180 |  | **-0.555** | 0.203 |  | **-0.551** | 0.208 |
|  |  |  |  |  |  |  |  |  |
| **Human resources for health** |  |  |  |  |  |  |  |  |
| Medical doctor |  |  |  | **-82.5** | 0.096 |  | **-84.7** | 0.095 |
| Public health nurse |  |  |  | **-9.40** | 0.078 |  | **-9.41** | 0.079 |
| Registered midwife |  |  |  | **10.5** | 0.227 |  | **10.3** | 0.237 |
| Barangay health worker |  |  |  | **0.452** | 0.415 |  | **0.488** | 0.399 |
|  |  |  |  |  |  |  |  |  |
| **Socioeconomic factor** |  |  |  |  |  |  |  |  |
| Urbanization level |  |  |  |  |  |  | **-0.240** | 0.821 |
|  |  |  |  |  |  |  |  |  |
| Obs |  | 201 |  |  | 201 |  |  | 201 |
| R-square |  | 0.723 |  |  | 0.733 |  |  | 0.7332 |

**Supplementary Figures**


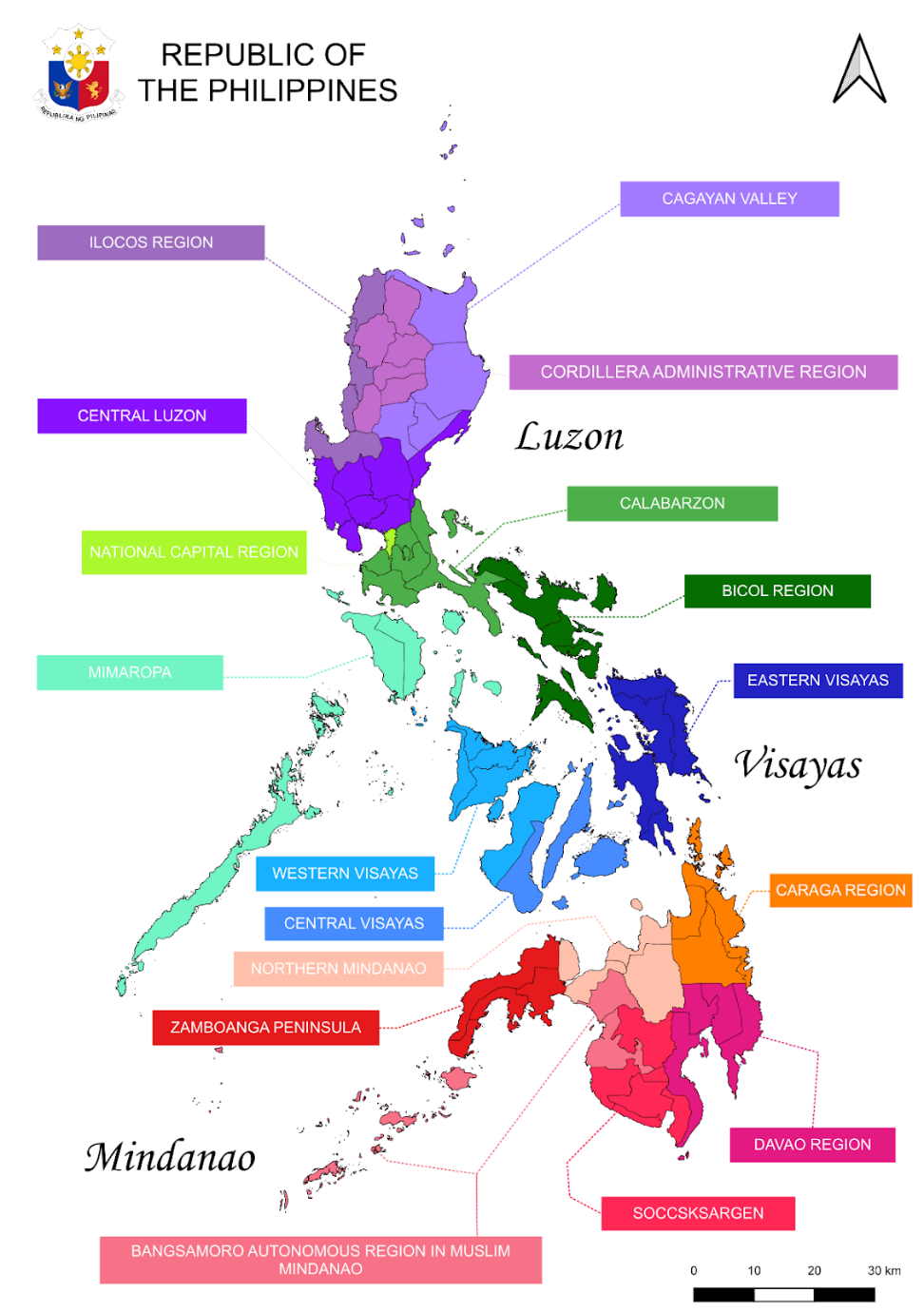


**Figure S1. Map of the Republic of the Philippines with its seventeen administrative regions. Generated from QGIS (version 3.34.0) with DIVA-GIS as base map.**


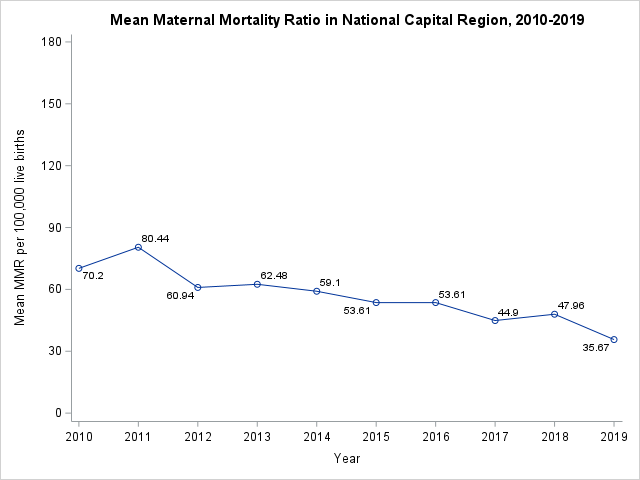

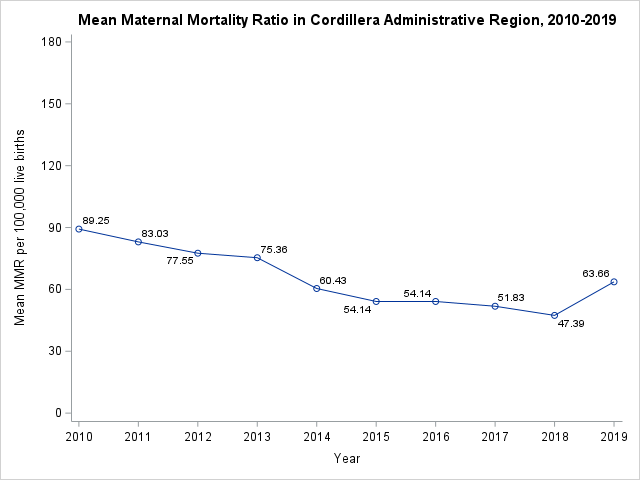

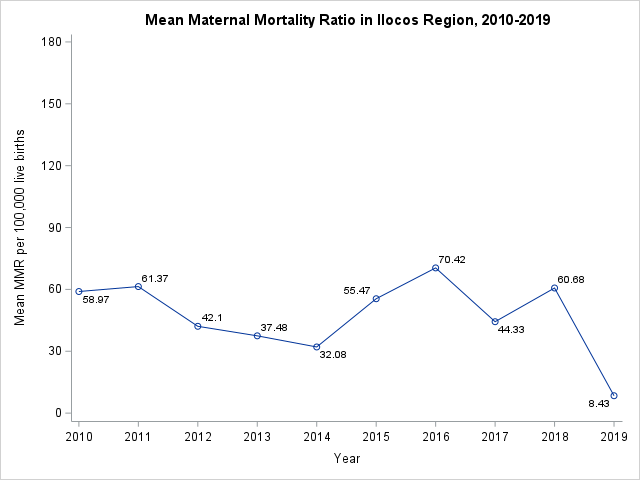

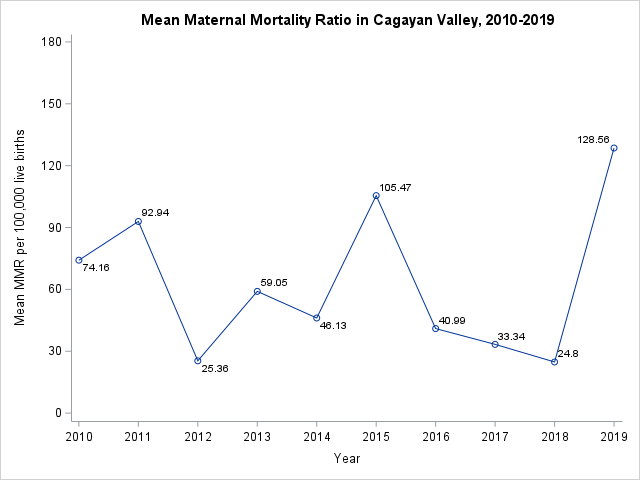

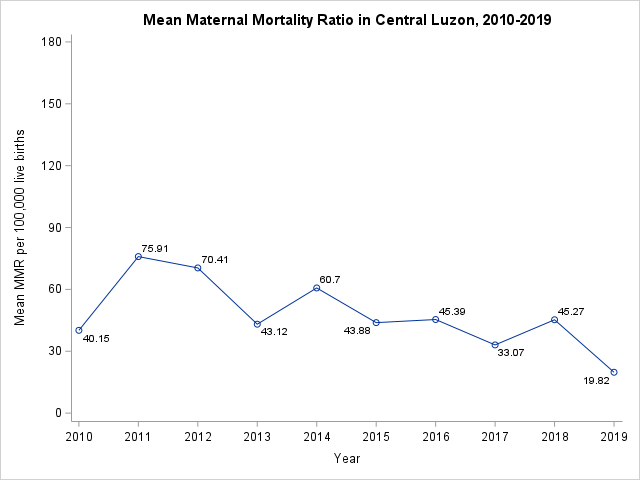

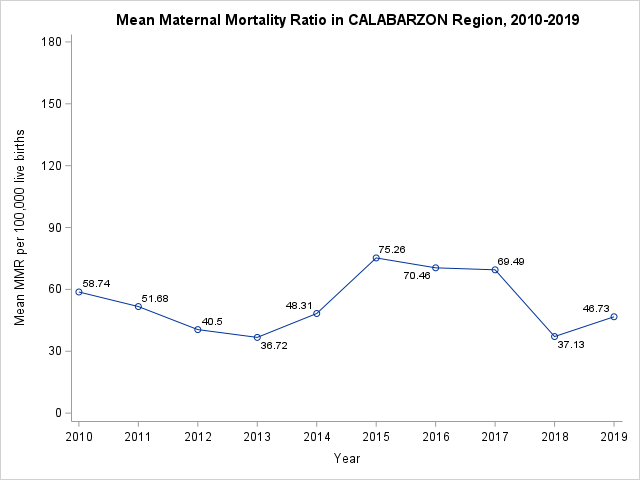

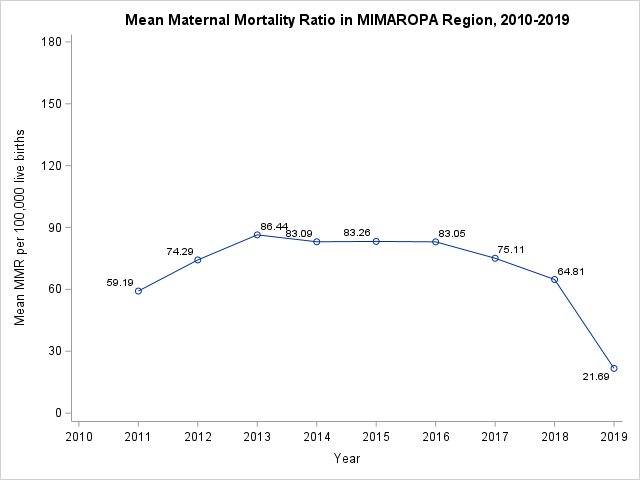

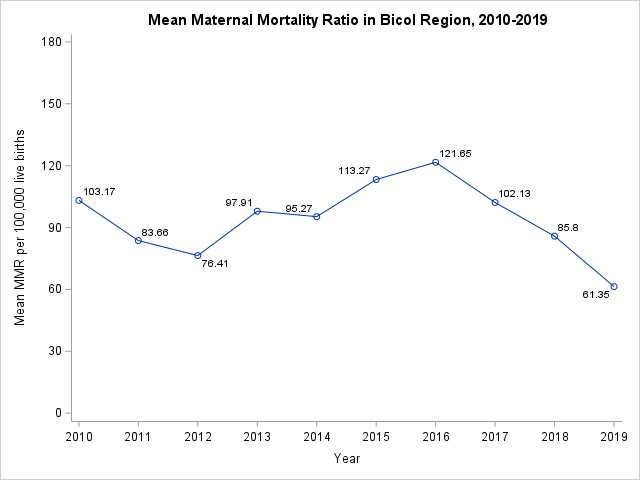


**Figure S2. Regional trends in maternal mortality ratio in Luzon, Philippines, 2010–2019**


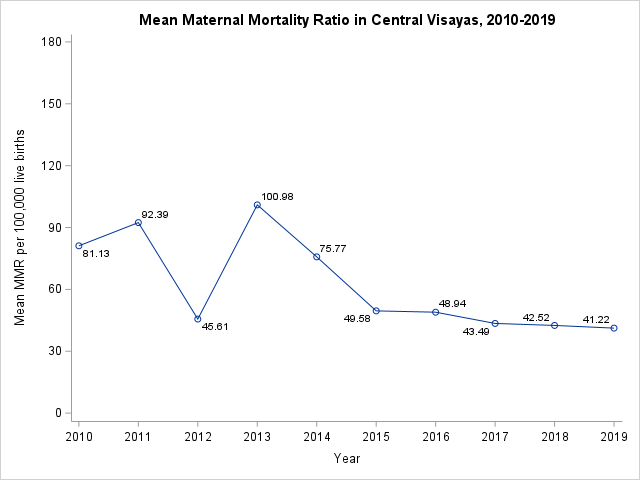

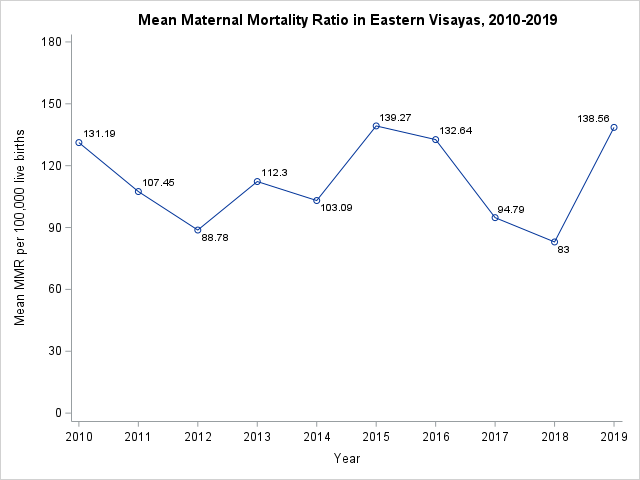

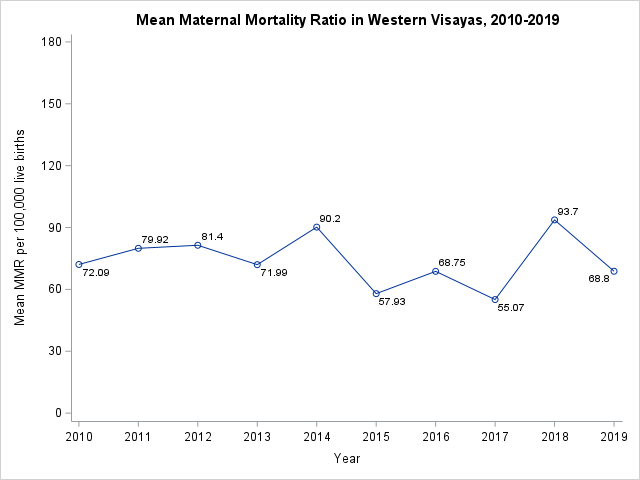


**Figure S3. Regional trends in maternal mortality ratio in Visayas, Philippines, 2010–2019**


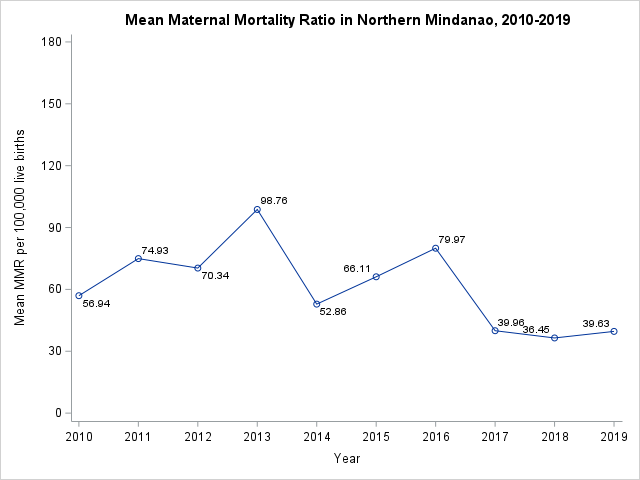

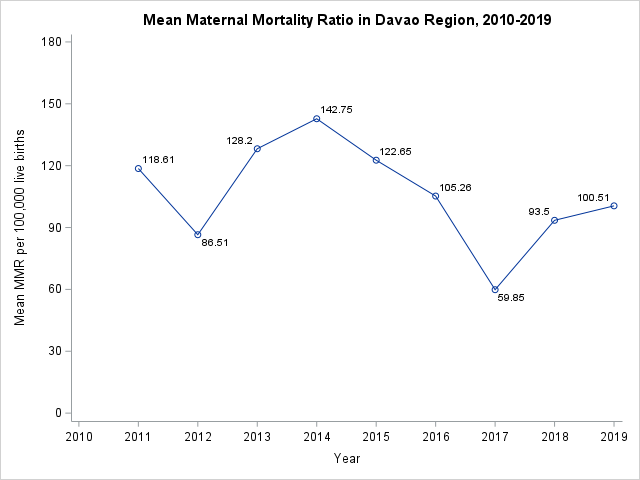

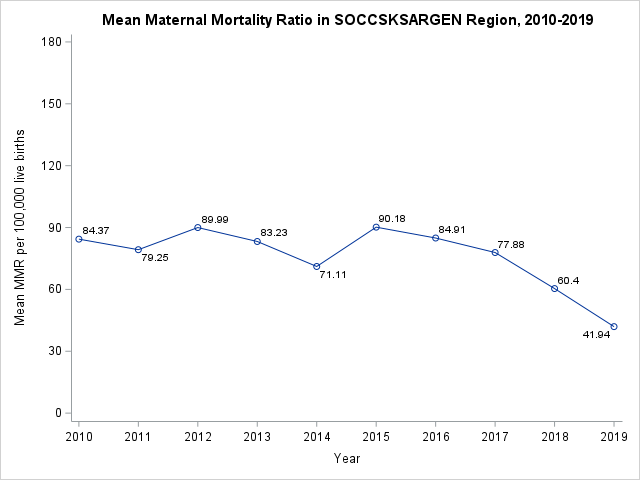

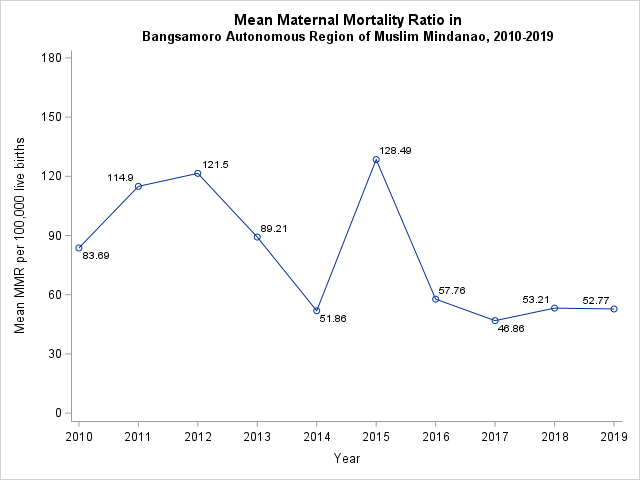

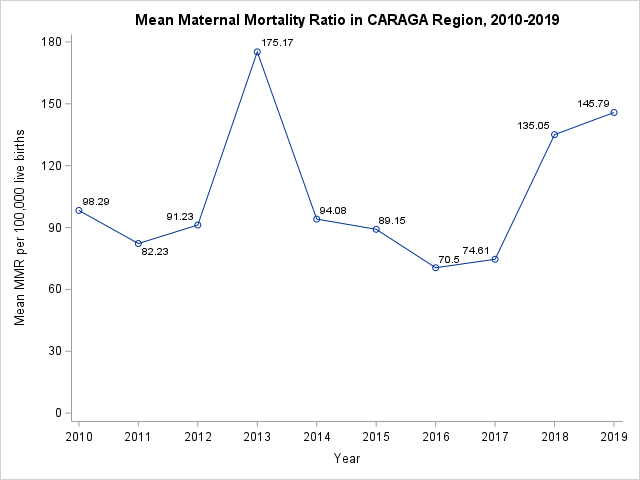

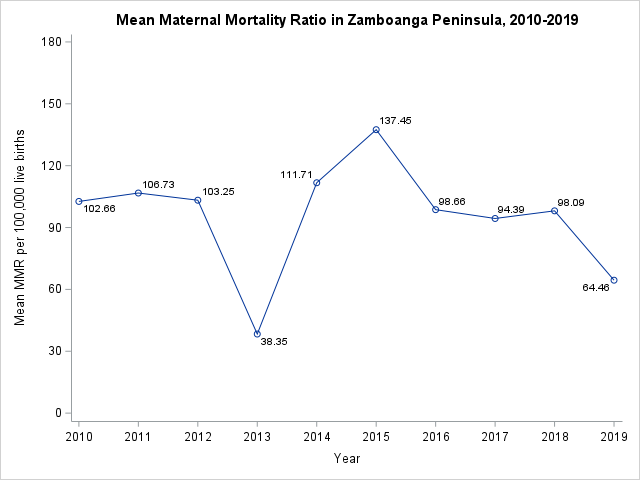


**Figure S4. Regional trends in maternal mortality ratio in Mindanao, Philippines, 2010–2019**
